# What we know and don't know about great ape cultural communication in the wild

**DOI:** 10.1002/ajp.23560

**Published:** 2023-10-12

**Authors:** Ammie K. Kalan, Robyn Nakano, Lindsey Warshawski

**Affiliations:** ^1^ Department of Anthropology University of Victoria Victoria British Columbia Canada

**Keywords:** bonobo, chimpanzee, gesture, gorilla, orangutan, Pan, Pongo, vocal

## Abstract

Following the first descriptions of culture in primates, widespread agreement has developed that the term can be applied to nonhumans as group‐specific, socially learned behaviors. While behaviors such as those involving extractive tool use have been researched intensively, we propose that behaviors that are more subtle, less likely to be ecologically constrained, and more likely to be socially shaped, such as cultural forms of communication, provide compelling evidence of culture in nonhuman primates. Additionally, cultural forms of communication can provide novel insights into animal cognition such as the capacity for conformity, conventionalized meanings, arbitrariness in signal forms, and even symbolism. In this paper we focus on evidence from studies conducted on wild great apes. First, we provide a thorough review of what exactly we do know, and by extension don't know, about great ape cultural communication. We argue that detailed research on both vocal and gestural communication in wild great apes shows a more nuanced and variable repertoire than once assumed, with increasing support for group‐specific variation. Second, we discuss the relevance of great ape cultural communication and its potential for illustrating evolutionary continuity for human‐like cultural attributes, namely cumulative culture and symbolism. In sum, a concerted effort to examine cultural forms of communication in great apes could reveal novel evidence for cultural capacities that have thus far been heavily debated in the literature and can simultaneously contribute to an improved understanding of the complex minds of our closest living relatives.

## Research Highlights

- Both vocal and gestural signals in great apes demonstrate some group‐specific variation with compelling evidence for cultural behaviors in the communicative domain.
- Cultural variation includes presence/absence of signals but also more nuanced variation in signal form or function.
- More cross‐site comparisons across and within great ape species are necessary to reveal the full extent and richness of cultural communication in our closest living relatives.

## Graphical abstract

Vocal and gestural communicative signals showing evidence for cultural variation in the wild.

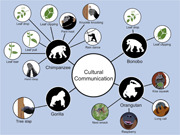

**Abbreviation**
AST: accumulative stone throwing

## 1 INTRODUCTION

Since the initial descriptions of “pre” or “sub‐culture” (Kawai, 1965; Kawamura, 1959) and then culture in primates (Whiten et al., 1999), there is now widespread agreement that the term culture can be applied to nonhumans, as group‐specific, socially learned behaviors (Whiten, 2021). In the last couple of decades, the field has focused on understanding the mechanisms that permit cultural variation to exist, such as innovation, conformity, teaching, and the various forms of social learning. More recently, descriptions of cumulative culture in humans have effectively re‐introduced a “cultural” boundary of sorts, again separating nonhumans from humans. Cumulative culture is defined as behaviors that become more complex or more efficient over generations and are beyond the scope of a single individual to invent on their own (Dean et al., 2014; Sasaki & Biro, 2017; Schofield et al., 2018; Tennie et al., 2009). While cumulative culture is argued to be uniquely human, in the last five years, multiple primatological studies have met the cumulative culture challenge head‐on, demonstrating both observational and experimental evidence to suggest that this cumulative culture boundary may in fact be a gradient where nonhuman primates may possess rudimentary forms (Boesch et al., 2020; Koops et al., 2022; Schofield et al., 2018). Nested within this debate of whether animals possess cumulative culture, we observe remnants of the “culture wars” reappearing (Langlitz, 2020) and thus are reminded of the need for compelling and robust evidence of culture if we are to truly understand this social phenomenon and its evolutionary origins in nonhuman primates. It is therefore necessary that we broaden our inquiry from the well‐studied, relatively easy to observe, conspicuous cultural behaviors, such as those involving tool use for extractive foraging, and shift our attention to the behaviors that are more subtle, less likely to be ecologically constrained, and more likely to be socially shaped, namely cultural forms of communication.

The majority of primate cultural behaviors described to date are in a foraging context, often involving the use of tools (Boesch, 2012; Whiten et al., 2001). Among these, some of the strongest evidence comes from nut cracking and termite fishing in chimpanzees (Boesch et al., 2020; Luncz et al., 2012; Pascual‐Garrido, 2019), seed extraction by orangutans (van Schaik et al., 2003), and food washing behaviors of Japanese macaques (Kawai, 1965; Schofield et al., 2018). Notably, many of the initial surveys of potentially cultural behaviors, collated from years of observations at long‐term field sites, emphasized socio‐communicative behavioral variation in taxa such as bonobos (Hohmann & Fruth, 2003), capuchins (Perry, 2011), chimpanzees (Whiten et al., 1999), orangutans (van Schaik et al., 2003), and more recently in gorillas (Robbins et al., 2016). However, in many cases these socio‐communicative behaviors did not become the focus of detailed follow up studies. Rather, extractive tool use behaviors were researched more intensively when present in a population. As mentioned above, extractive tool use behaviors are amenable to detailed studies due to their conspicuous nature, the artefacts left behind, and relatively predictable occurrence, i.e., the food item being extracted. This early bias in primate cultural studies inadvertently denoted socio‐communicative behavioral variation within a species as less pertinent to the study of primate cultures. This is surprising given that it is indeed in the communication domain where we find some of the most compelling evidence for cultures in other taxa, such as songbirds (Aplin, 2019) and whales (Whitehead & Rendell, 2015, but see also Mercado, 2022). It is perhaps due to the historically held perception that primate vocalizations are inflexible and involuntary (Seyfarth & Cheney, 2010) that all other forms of communication were considered to be as well, and therefore not worth pursuing through a cultural lens. However, in the 90s, there were multiple promising studies investigating the social aspects of great ape long distance calls and potential vocal dialects (Hohmann & Fruth, 1995; Mitani, 1985; Mitani & Stuht, 1998; Mitani et al., 1992). Similarly, investigations into great ape gestural signals highlighted the potential for flexibility and innovation in the wild (Boesch, 1995; Sugiyama, 1981). Building on these early studies, detailed research on both vocal and gestural communication in wild great apes now shows a more nuanced and variable repertoire than once assumed, with increasing support for socially‐shaped, group‐specific variation.

The significance of cultural forms of communication in nonhuman apes is paramount to our understanding of the degrees of separation between human and nonhuman cultures. Because communication is inherently social, requiring at minimum two individuals—the signaler and the receiver—communicative behaviors are more prone to being shaped by social environments, compared to foraging behaviors, and thus by social learning. Moreover, their relative (although not complete, see signals involving objects below) independence from the ecological environment makes them exceptional examples of nonhuman culture. This is further supported by the lack of a tangible end‐product, such as a food item, which may simultaneously serve as a reward and a learning stimulus. Hence, cultural communicative behaviors may be expected to rely more on high fidelity learning given that subtle nuances in actions or movements can be informative when it comes to signaling interactions. We may therefore predict greater *know‐how* (instead of *know‐what*) social learning (Tennie et al., 2020) relative to extractive foraging behaviors. In this manner, cultural forms of communication may be expected to be more human‐like in their potential for exhibiting greater conformity, conventionalized meanings, arbitrariness in signal forms, and even symbolism (Boesch, 2012; Dean et al., 2014; Henrich & Tennie, 2017).

Here, we present a review of the communicative vocal and gestural signals present in our closest living relatives, the great apes, that demonstrate evidence for group‐specific, socially learned variation in the wild. This paper (1) summarizes what exactly we do know, and by extension don't know, about great ape cultural communication by cataloging behaviors for each great ape, and (2) examines its relevance and potential for illustrating evolutionary continuity for human‐like cultural attributes, namely cumulative culture and symbolism. More specifically, we describe the evidence thus far for considering vocal and gestural signals as cultural in wild great apes, highlighting for what and where we need further empirical data. Due to the breadth of research on the topic of primate cultures, this review concerns itself with evidence from studies conducted in the wild, and focuses on describing observations of putative cultural communication behaviors. Although we recognize the valuable research that studies in captive settings provide to the study of animal culture, especially with regard to understanding cultural transmission and social learning mechanisms, we refrain from delving into captive studies given the large corpus of captive research on ape communication. In general, the behaviors included in this review have not been unequivocally designated as cultural in the literature due to uncertainty regarding learning and transmission mechanisms hence additional research will be necessary. However, we take a conservative approach and include any communicative behaviors as potentially cultural that exhibit group‐specific variation, are not universally observed in the species, and whose occurrence has not been attributed to a specific environmental resource. Given recent calls for incorporating animal cultures into conservation efforts (Brakes et al., 2019; Carvalho et al., 2022; Kühl et al., 2019), we err on the side of caution before eliminating a behavior from a species' potential cultural repertoire. This review therefore represents a critical first step, where it is necessary to identify those behaviors that demonstrate promise for cultural variation so that additional research and experiments can be designed to provide more conclusive evidence of their cultural nature.

## 2 EVIDENCE FOR GREAT APE CULTURAL COMMUNICATION IN THE WILD

Differences in communicative behaviors between great ape populations can occur in form, structure, function, and in presence/absence across populations (Pika & Deschner, 2019; Whiten et al., 2001). Below, we review evidence for cultural variation in communication for each of the four major great ape taxa by focusing on evidence for group‐specific traits in the two dominant modes of communication: vocal (i.e., acoustic signals produced by the vocal chords or movement of the lips/tongue) and gestural.

### 2.1 Chimpanzees (*Pan troglodytes*)

#### 2.1.1 Vocal

##### Pant hoots

Population variation in chimpanzee vocalizations has primarily focused on the pant hoot—a complex, multicomponent, long‐distance call that is comprised of up to four distinct phases: introduction, build‐up, climax, and let‐down (Goodall, 1986; Mitani et al., 1992). The pant hoot generally functions to coordinate movement and facilitate cohesion between group members, but there are notable acoustic differences in the individual phases of pant hoots between populations which have been argued to represent socially learned variation. In their seminal study, Mitani et al. (1992) looked at acoustic differences between two populations of eastern chimpanzees in Tanzania, those inhabiting the Mahale Mountains and those living in Gombe Stream National Park. The study revealed subtle differences between the two communities' calls. Compared to Gombe chimpanzees, the build‐up portion of Mahale individuals' pant hoots exhibited a faster rate and shorter acoustical elements. Additionally, Mahale chimpanzees' climaxes were found to be both broader‐band and higher pitched. The authors suggested it was unlikely that the differences were due to genetic, anatomical, temporal, or contextual causes. As such, Mitani et al. (1992) ultimately proposed that the differences were dialectical, and that variation in pant hoots may reflect “selective reinforcement” wherein over time vocal learning has resulted in these vocalizations becoming community‐specific, which in turn raises the possibility that such differences may be cultural. However, genetic or environmental differences were difficult to completely rule out in this study, particularly how variation in habitat and topography could influence call rate and structure. Unlike Mitani et al. (1992), Crockford et al.'s (2004) analysis of four, including three neighboring, communities of western chimpanzees in the Taï forest of Côte d'Ivoire was able to definitively rule out genetic associations for acoustic differences between the different communities' pant hoots by including genetic data. Results showed a discernable difference between three neighboring communities' pant hoots. Additionally, the fourth, geographically‐distant community's acoustic parameters discriminated poorly from the contiguous communities' calls, suggesting that differences in pant hoots are learned as opposed to genetically determined (Crockford et al., 2004). Importantly, Crockford et al. (2004) ruled out environmental differences given that all communities lived in the same continuous forest, adding credence to the notion that variation in chimpanzee pant hoots may in fact be cultural. However, other authors have highlighted the low sample size of individuals per community that were included in the Crockford et al. study (Desai et al., 2022) therefore repetition of this research with a greater number of individuals, ideally of both sexes as well, is needed. Overall, despite pant hoots being a universal vocalization in chimpanzees that is likely hard‐wired, current research suggests that the final acoustic form of the call demonstrates subtle population differences indicating some social fine‐tuning via *know‐how* learning.

Population differences specific to the structural features of pant hoot phases have also been reported in the literature. Arcadi (1996) compared chimpanzees living in Kibale National Park, Uganda with published pant hoot data from Mahale and Gombe chimpanzees (see Mitani et al., 1992) and found notable qualitative differences between the two communities. Specifically, Kibale chimpanzees rarely uttered a build‐up phase in their pant hoots, whereas Mahale and Gombe chimpanzees typically did include a build‐up phase (Arcadi, 1996). However, with their follow‐up assessment of additional recordings, Mitani et al. (1999) argued that Kibale chimpanzees do indeed include a build‐up portion in their pant hoots, but that other acoustic differences between Kibale and Mahale chimpanzees were notable. The authors showed that Kibale chimpanzees' build‐up phases were characterized by having longer elements over shorter periods, and at slower rates (Mitani et al., 1999). In contrast to Arcadi's (1996) initial conclusions, Mitani et al. (1999) suggested that variation between the two sites may be due to ecological differences in habitat and anatomical differences in body size. That their reassessment came to different conclusions than Arcadi's (1996) original study may be due to the fact that a number of the Kibale vocalizations were taken from nonhabituated individuals where recordings were made at greater distances and may have been susceptible to interference. More recently, Desai et al. (2022) reported differences in the climax phases between two neighboring communities of Gombe—Kasekela and Mitumba—compared to the geographically distant Kanyawara community. While the authors found that individual differences were more prominent than group differences, acoustic variation between the three populations' climax phases was evident, further supporting group‐specific variation in pant hoots while ruling out genetic and habitat differences (Desai et al., 2022).

While there is considerable evidence supporting variation in the acoustic properties of chimpanzee pant hoots, recent research further suggests population variation in the combination of pant hoots with other calls. Specifically, pant hoots are combined with pant grunts, a species‐typical greeting call, to create what is referred to as a collective greeting hoot sequence. However, the order of the call combination differs across populations, with Taï forest chimpanzees using a pant hoot‐pant grunt sequence whilst chimpanzees of the Budongo forest in Uganda use a pant grunt‐pant hoot sequence (Girard‐Buttoz et al., 2022). This variation in call order preference is unlikely to be a result of genetic or environmental variation given that both call types are present in both populations. Variation in call combinations therefore represent a promising form of population‐variation which may provide further insight into cultural communicative forms in great apes.

Pant hoots have also been proposed to serve different functions such as announcing food location (Wrangham, 1977), advertising social status (Clark & Wrangham, 1994), recruiting social allies (Mitani & Nishida, 1993), and even referential meaning (Uhlenbroek, 1995). A within‐community analysis of pant hoots produced by Budongo forest chimpanzees, similarly found support for acoustic variation associated with different behavioral contexts (Notman & Rendall, 2005). However, these behavioral functions have not been investigated as potentially varying across populations although there may be scope to do so in the future given the large number of pant hoot recordings and cross‐site databases available.

##### Food‐associated calls

Aside from pant hoots, there is some research to suggest potential variation across populations with regard to chimpanzee food‐associated calls. When foraging, chimpanzees produce acoustically distinct and highly variable vocalizations that grade from low‐pitched grunts to higher‐pitched barks (Goodall, 1986). These food‐associated calls are only produced for approximately half of all feeding events, suggesting they are not solely an emotional or physiological response to the presence of food (Kalan et al., 2015; Slocombe, et al., 2010). Studies from Taï have shown subtle acoustic variation in food calls associated with the size of food patches, whereby chimpanzees produced lower‐pitched food calls for larger *Nauclea* fruit trees (Kalan et al., 2015). However, similar fine‐scaled acoustic analyses have not been repeated for other communities. Of particular interest would be food call analyses for the same food species in different and neighboring chimpanzee communities. The function, rather than form, of food‐associated calls has been better studied in the wild, and suggests there may be variation across communities with regard to audience effects. In the Taï forest, there was general support for food calls serving a recruitment function as chimpanzees were more likely to join a feeding event after food calls had been produced (Kalan & Boesch, 2015). Taï chimpanzees were also more likely to food call when there were more males present in their party, but also when estrous females were nearby (Kalan & Boesch, 2015). This is in contrast to results from Slocombe et al. (2010) who reported that the presence of estrous females did not influence food‐associated calling in the Sonso chimpanzee community of Budongo, though they only examined presence of estrous females in the party, not those who might have been nearby, as in the Kalan and Boesch (2015) study. However, Slocombe et al. (2010) did find a clear influence of the presence of social male allies increasing the probability for males to produce a food call, which is in line with the results from Taï (Kalan & Boesch, 2015) and Kanyawara (Fedurek & Slocombe, 2013). A recent study of the Sonso community found further evidence that low‐ranking males were most likely to produce food‐associated calls when high‐ranking males arrived at a food patch, indicating that food‐associated calls serve to reduce aggression pertaining to feeding competition (Bouchard & Zuberbühler, 2022). In the Kanyawara community, food‐associated calls were more likely to prolong the duration of a feeding event, perhaps facilitating cofeeding with social allies (Fedurek & Slocombe, 2013). So, although there is potential variation in the function of food‐associated calls across communities, clear evidence of population variation has been hindered due to the different methods and analytical frameworks used in all of these individual studies. With a systematic, unified approach, data on food‐associated calls could be more efficiently compared across sites to provide an additional avenue of investigation into cultural communication in chimpanzees.

#### 2.1.2 Gestural

Communicative gestures are defined in contrast to other behavioral traits by their increased intentionality and flexibility; these gestures are performed with the goal of influencing the recipient's behavior, and the function of an action may differ between instances (Tomasello & Call, 2019). In contrast to vocalizations, gestural communication, and variation in gestures between chimpanzee populations is comparatively understudied (but see Pika & Deschner, 2019). Nevertheless, there is compelling evidence demonstrating variation in gestures, including those involving object modification or tool use.

##### Gestures with objects or tools

Leaf‐modifying gestures have recently been described as forming gestural dialects in neighboring groups of chimpanzees in the Budongo forest (Badihi et al., 2023). The detailed analysis of Badihi et al. (2023) supports decades of long‐term observations of chimpanzee leaf‐clipping behavior across sites from multiple individual studies. Leaf‐clipping was first reported by Nishida (1980) in the Mahale K group, and is described as when: “a chimpanzee picks off one to five stiff leaves, grasps the petiole between the thumb and the index finger, repeatedly pulls it from side to side while removing the leaf‐blade with the incisors, and thus bites the leaf to pieces. In removing the leaf‐blade, a ripping sound is conspicuously and distinctly produced. When only the mid‐rib with tiny pieces of the leaf‐blade remains (and the mid‐rib often resembles a tooth‐pick), it is dropped and another sequence of ripping up a new leaf is often repeated" (p. 117). Leaf‐clipping has since been reported in various chimpanzee communities across Africa with notable variation in contexts and behavioral outcomes. Nishida (1980) found that K group adult and adolescent males, as well as estrous females, engaged in leaf‐clipping to solicit copulation. Watts (2007) as well as Hobaiter and Byrne (2014) describe leaf‐clipping primarily to initiate copulation in Budongo and Ngogo chimpanzees in Uganda, respectively. Further variation is supported by Sugiyama (1981) who found that chimpanzees in Bossou, Guinea performed leaf‐clipping in playful contexts and while frustrated. Similarly, Taï chimpanzees were observed to leaf‐clip during times of frustration, but they also incorporated the behavior into drumming displays wherein they leaf‐clipped before emitting pant hoots (Kalan & Boesch, 2018). Similarily, leaf‐clipping during displays was also observed in Cantanhez chimpanzees of Guinea‐Bissau who leaf‐clipped while buttress drumming (Bessa et al., 2022). In a comparison of leaf clipping and other behaviors in three neighboring communities of the Taï forest, Luncz and Boesch (2015) described group‐specific variants in the presence of a gesture and/or its meaning, thereby illustrating a strong case for cultural communication. A recent theoretical analysis including auditory, object modifying gestures observed in Taï chimpanzees, namely leaf clipping and knuckle‐knocking, further argues for symbolic signal usage based on the socially‐derived meanings of each gesture and their apparent arbitrariness with respect to signal form and meaning (Cissewski & Luncz, 2021). The method by which chimpanzees modify the leaf during leaf‐clipping, and during other leaf‐modifying gestures, has been relatively understudied until recently (Badihi et al., 2023) but is critical to illuminating cultural variation given that subtle variations in actions must be socially learned if they are to give rise to group‐specific dialects.

Buttress drumming is another gesture involving an object, a tree, that can occur in a number of forms: the drummer may stand on the ground and use their hands to hit both sides of a buttress root, they may stand atop the buttress root and hit it with their hands and/or feet, or they may hit the buttress with their hands while traveling past it (Arcadi et al., 1998). While buttress drumming is a universal chimpanzee behavior it has also been found to vary across populations. Arcadi et al. (1998) reported significant interpopulation variation in buttress drumming between Taï and Kanyawara chimpanzees, with individuals at Kanyawara tending to drum more often without pant hooting than Taï chimpanzees. However, when they did incorporate vocalizations into drumming displays, Kanyawara chimpanzees drummed later on during the pant hoot. These patterns, although interesting, could simply reflect subspecies differences and would therefore be strengthened with additional evidence from studies investigating neighboring or within subspecies variation. There has also been a reported instance of buttress drumming patterns by a particular adult male in the Taï forest uniquely conveying direction of travel to other group members in an intricate, symbolic‐like fashion with the behavior unfortunately ending with the death of this male (Boesch, 1991). Although the behavior failed to transmit within the group, one would predict that there must have been sufficient social learning to permit the meaning of the signal to have been understood by other group members during the lifetime of that adult male (see also orangutan long‐calls described below). In general, these studies highlight the nuanced variation in buttress drumming patterns that may provide further insight into potential group‐specific and thus cultural variation.

A potential variant of buttress drumming is the relatively recently documented behavior in chimpanzees, accumulative stone throwing (AST; Kühl et al., 2016). AST has only been documented in four chimpanzee communities in West Africa and is otherwise absent from any long‐term, habituated chimpanzee field sites (Kühl et al., 2016). Using remote camera‐trap devices, chimpanzees have been recorded throwing rocks at the same trees repeatedly, in conjunction with their long‐distance pant‐hoot vocalization, and sometimes drumming on the buttresses or trunk of the tree (Kühl et al., 2016). The rocks are thrown in an aimed fashion at the tree's trunk, buttress roots, or into the hollow cavity of the trunk, resulting in the trees becoming visually marked with scars from the repeated impacts of the rock (Kalan et al., 2019). Given that rock and tree availability do not appear to explain the occurrence of AST in these communities (Kühl et al., 2016), a social explanation has been put forth whereby AST has emerged as a cultural phenomenon in these communities. As with many of the behaviors described in this article, longitudinal data may help us to study the social learning and transmission of this behavior within these communities.

##### Gestures without objects

One of the most well‐studied gestures that exhibits cultural variation is the grooming hand‐clasp, which can be considered a postural or tactile‐gesture. It is defined by McGrew and Tutin (1978) as when: each of the participants simultaneously extends an arm overhead, and then either one clasps the other's wrist or hand, or both clasp each other's hand. Meanwhile, the other hand engages in social grooming of the other individual's underarm area revealed by the upraised limb, using typical finger movements. In doing this, the two chimpanzees sit facing each other on the ground in a symmetrical configuration. Either both raise their right arms and groom with their left, or vice‐versa (p. 238). In their analysis of 65 behaviors across six long‐term field sites, Whiten et al. (1999) found the ‘grooming hand‐clasp’ to be habitual for chimpanzees in Taï, customary among M and K groups at Mahale, but absent for Gombe, Bossou, and Budongo chimpanzee communities. Nakamura and Uehara (2004) also reported observations of the grooming hand‐clasp among Kanyawara and Ngogo chimpanzees, as well as in Kalinzu chimpanzees in Uganda, and those inhabiting Lope National Park, Gabon. More recently Pika and Deschner (2019) reported hand clasping in the Rekambo chimpanzee community in Loango National Park, and Gabon and Piel et al. (2017) reported the behavior in the Issa chimpanzee community in Tanzania. In addition to the grooming hand‐clasp being present and absent in various communities, its form also varies across field sites. Mahale K group chimpanzees perform a palm‐to‐palm hand‐clasp, whereas Mahale M group individuals perform a wrist‐to‐wrist hand clasp. Interestingly, an adult female who immigrated from the K to M group was seen adopting the groups' wrist‐to‐wrist custom, suggesting that new group members adopt their new groups' tradition (McGrew et al., 2001). However, a detailed analysis of the Kanyawara chimpanzees has shown that individuals vary in hand‐clasp and high‐arm grooming styles, primarily adopting the style used by their mother while lacking a clear group‐level preference (Wrangham et al., 2016). Hence, further research is needed to investigate the modes of transmission and social learning maintaining grooming hand‐clasp traditions across populations.

**Figure 1:**
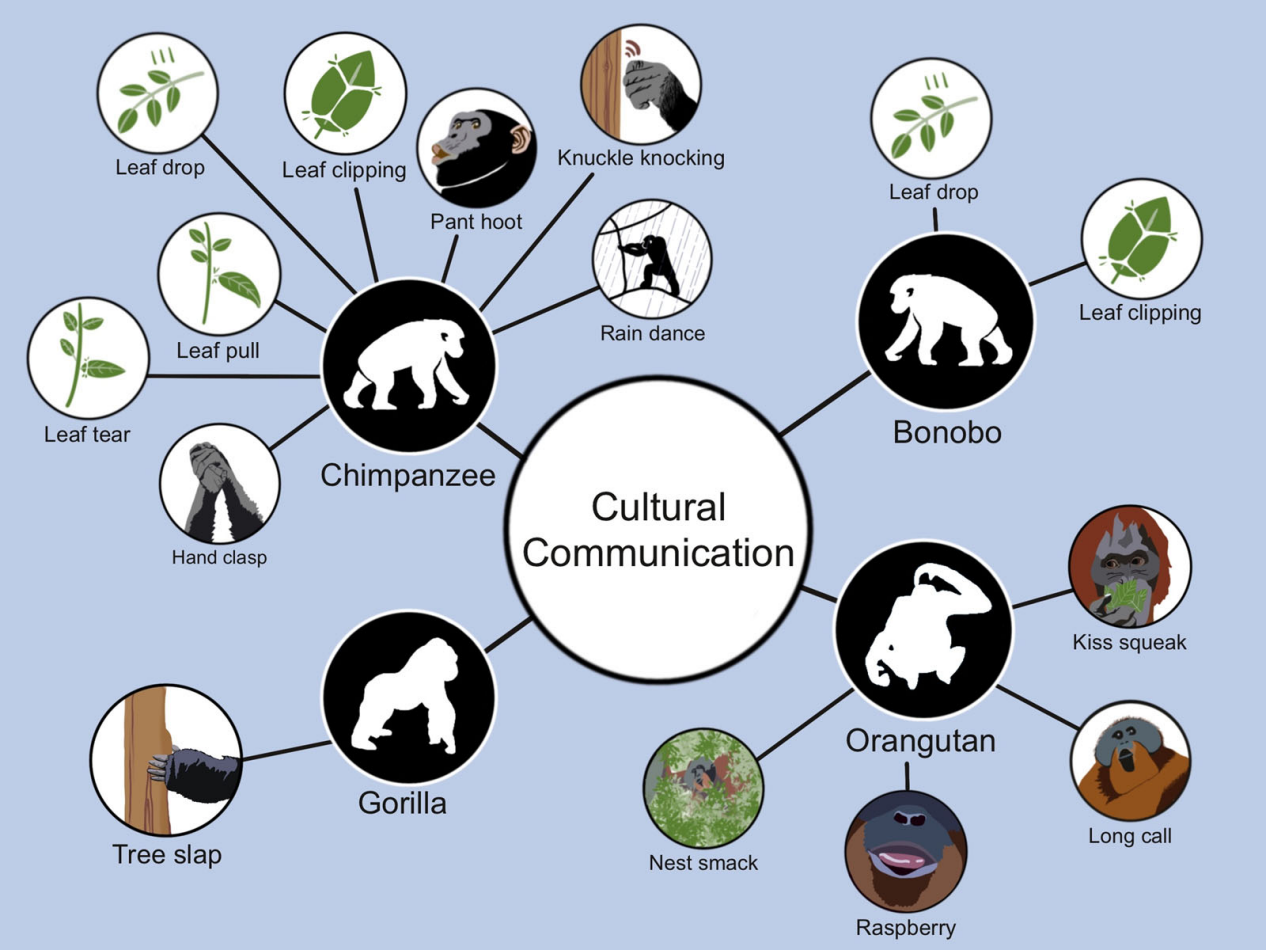
Forms of vocal and gestural communication signals in the four major taxa of great apes that show strong evidence for cultural, group‐specific variation in the wild (see also Table 1).

Another gesture exhibiting potential variation between populations is the rain dance, where adult males perform vigorous displays in response to the start of heavy rain (Whiten et al., 2001). First recorded by Goodall (1971), these displays are observed in Taï, Budongo, Gombe, Kibale, and Mahale chimpanzees whilst absent in Bossou (Whiten et al., 1999). In addition, the type or form of the rain dance is suggested to vary. Some observers described chimpanzee rain dances as being fast and noisy, including loud pant hoots for example, whilst others emphasized slow and silent displays (Whiten et al., 2001). Kalinzu chimpanzees have also been observed engaging in the behavior, with adult males rushing to the tops of trees, vocalizing, and breaking branches during periods of heavy rain (Hashimoto, 1998). The rain dance, and other rhythmic movements induced in response to sound, has been suggested to be the prerequisite for music and dance (Hattori & Tomonaga, 2020), and could provide an example of proto‐ritual (Tennie & van Schaik, 2020) although these claims remain largely untested. More importantly, other than vague descriptions in the literature, the rain dance has never been systematically analyzed nor studied. It therefore remains difficult to determine which aspects of this display are different from the general male‐display behavior observed in wild chimpanzees (Clark & Wrangham, 1994; Goodall, 1986; Kalan & Boesch, 2018) other than the fact that it appears to be triggered by the occurrence of hard rains or waterfalls (Figure 1).

### 2.2 Orangutans (*Pongo sp*)

#### 2.2.1 Vocal

Like chimpanzees, orangutans also exhibit vocal variability between populations. The most studied orangutan vocalization, the ‘long‐call’, is a multi‐unit call consisting of a “bubbly‐like” introduction which is then followed by a sequence of “barks” or “pulses,” and concludes with a series of “sighs” (Setia & van Schaik, 2007). The long‐call is most often emitted by males, and generally functions to repel other, lower‐ranking males, and attract higher‐ranking males, as well as sexually receptive females (Mitani, 1985; Setia & van Schaik, 2007). These vocalizations have been found to vary acoustically across different geographic locations. Delgado et al. (2008) reported differences in call duration, speed, number, and duration of pulses, as well as in fundamental frequency between Bornean (*Pongo pygmaeus*) and Sumatran (*Pongo abeli*) orangutans. While the authors acknowledge that such differences may be genetic or due to differing ecologies or habitats of the two species (Delgado et al., 2008), a later study by Krützen et al. (2011) found putative cultural traits, including long‐calls, are not well explained by genetic or environmental factors, despite considerable genetic differences between populations.

In addition to regulating distances between males and sexually active females, long‐calls of flanged males in Suaq Balimbing on the island of Sumatra have also been shown to convey travel direction. Female listeners adjusted their ranging behavior in accordance with these vocalizations and tended to remain within earshot of calling males, whereas male listeners tended to increase the distance between themselves and the callers (van Schaik et al., 2013). Whether long‐calls in other orangutan populations on Sumatra or Borneo serve similar functions has yet to be investigated. Therefore, if this particular function of long‐calls in Suaq Balimbing males can be considered cultural remains to be seen but is reminisicent of the single case of travel direction being associated with the chimpanzee pant hoot (Boesch, 1991). Overall, these findings serve as an important starting point for future investigations into the varying function(s) of orangutan long‐calls and long‐distance vocalizations of other apes as well.

There is also geographic variation described in the vocal repertoire of wild orangutans in terms of specific vocalizations being present in some populations, while being absent in others. The orangutan's “raspberry” vocalization is produced by pushing air through the lips creating a spluttering sound (van Schaik et al., 2003). It is typically associated with nest building, but has only been observed in certain populations. According to van Schaik et al. (2003), it is habitual among Lower Kinabatangan orangutans on Borneo who raspberry just before nesting, and customary among Suaq Balimbing orangutans on Sumatra who raspberry during the final phase of nesting. Moreover, raspberry vocalizations are altogether absent in four other orangutan populations, with no clear explanation for habitat differences or genetic differences accounting for this geographic variation (Krützen et al., 2011; van Schaik et al., 2003; Wich et al., 2012). Wich et al. (2012) also reported both presence and absence of discrete call types, as well as acoustic variation in calls, between five geographically dispersed populations. Similar to van Schaik et al. (2003), Wich et al. (2012) found that Sumatran Suaq and Bornean Sabangau orangutans raspberry regularly during the final phase of nest building, whereas Bornean Tuanan individuals produce “nest smacks” when nesting. Conversely, nesting vocalizations are absent among Sumatran Ketambe and Bornean Sungai Lading orangutans, further demonstrating evidence that vocal variation in orangutan populations goes beyond species differences and cannot be explained by genetic variation (Wich et al., 2012). Overall, these studies suggest that orangutans possess vocal cultures wherein calls are invented and spread within a group via social learning.

##### Kiss‐squeak

The “kiss‐squeak,” which is considered universal among all orangutan populations that have been studied until now, functions as an alarm call. The sound is produced without engaging the vocal chords by taking air in sharply via pursed lips (Hardus et al., 2009; Lameira et al., 2013). Hardus et al. (2009) found that orangutans in Tuanan, Borneo kiss squeak with their hand and/or leaves positioned against their lips. This variation produces a lower frequency, and because frequency correlates with body size, this behavioral variant gives the impression of a larger‐than‐actual body size (Hardus et al., 2009). Interestingly, this aided kiss squeak is most often emitted when there is a perceived threat nearby, leading the authors to posit that Tuanan orangutans deceive potential predators by engaging in the aided kiss squeak (Hardus et al., 2009). Conversely, kiss squeaks among orangutans of the same subspecies at Cabang Panti on Borneo are just as common, but the hand‐aided variant is extremely rare, with the leaf‐aided variant being more common. However, unlike the Tuanan orangutans, the leaf‐aided kiss squeak does not portray a larger body size, nor is it associated with predatory threats (Lameira et al., 2013).

#### 2.2.2 Gestural

The gestures of orangutans have been only recently studied in the wild despite decades of work on captive populations, showing thus far no evidence for group‐level or population variation (Fröhlich et al., 2021). The gestures of orangutans may be more difficult to study due to their arboreal nature, although there are a number of gestures involving objects such as “shake object” or “present object” that may be especially interesting to focus on for population variation. These object gestures may have the potential to invite greater variability due to the myriad of ways that an individual can interact with an external object, as seen in the leaf‐modifying dialects of chimpanzees described above (see also discussion on auditory gestures in Part 3).

Given their extensive communicative repertoire (Delgado et al., 2008; Wich et al., 2012), it is likely that future research will inform us of even more vocal and gestural variability. Indeed, the Sumatran orangutans, which appear to be more socially gregarious than their Bornean counterparts, display higher degrees of vocal innovation (Lameira et al., 2022); therefore, examining variation among multiple populations of the same subspecies may be most promising for future research into cultural variation in the communicative domain.

### 2.3 Gorillas (*Gorilla sp*.)

#### 2.3.1 Vocal

Currently, of all the great apes, we have the least amount of support for communicative variation in gorillas (Robbins et al., 2016). Nevertheless, there is compelling evidence for behavioral differences within and between gorilla species (Robbins et al., 2016).

In their analysis of behavioral variation among three western gorilla (*Gorilla gorilla*) and two mountain gorilla (*Gorilla beringei*) sites, Robbins et al. (2016) identified vocal and gestural variation between groups. For mountain gorillas, the “raspberry,” similar to what is described for orangutans earlier, was found to be habitual among Karisoke gorillas in Rwanda, while also present in Bwindi, in Uganda, but completely absent at all three western gorilla sites (Robbins et al., 2016). The raspberry is the only vocal signal considered potentially cultural in gorillas but requires further investigation of within‐species variation in mountain gorillas. Interestingly, the raspberry is considered to be a cultural signal in orangutans and has also been reported occurring during grooming in a single group of wild chimpanzees, the Ngogo chimpanzees (Pika, 2014). Hence, this particular signal appears to demonstrate some flexibility across wild great apes in general.

#### 2.3.2 Gestural

There are five gestures highlighted by Robbins et al. (2016) that exhibit within and between species variation in gorillas. Two of the gestures are found only in one species, such as tapping head with hand (mountain gorillas) or hand clapping (western gorillas, see also [Kalan & Rainey, 2009]) while the other three show some variation across groups that is, not split by species. For example, tree slap, where the hands are used to beat against a tree similar in manner to a chest beat (gorilla universal behavior), has not been observed in the Karisoke mountain gorillas in Rwanda but has been seen at all other gorilla sites. The other two gestures include signals incorporated as parts of a display: pseudo‐feeding and splashing with water (Robbins et al., 2016). In a display similar to the chimpanzee rain dance, western gorillas of Bai Hokou in the Central African Republic and Mondika, which straddles the border between Central African Republic and the Republic of the Congo, have also been observed beating their chests and displaying as it starts to rain. Robbins et al. (2016) classified the gorilla rain dance as a rare behavior, absent at most sites. Overall, there is promising variation in some communicative signals both between and within species that merit further investigation but other than perhaps the tree slap, the evidence is currently inconclusive.

Beyond presence/absence of signals, there is also the potential for variation in the contexts or functions of these signals within a gorilla group. It has been proposed that auditory gestures, like hand clapping and chest beating, may serve different functions in different groups, adding another layer of potential socially learned, cultural variation to gorilla communication in line with what has been thus far documented in chimpanzees. Salmi and Muñoz (2020) examined the occurrence of chest beating and hand clapping in detail for the Mondika western lowland gorillas noting context and age sex class of the signaler, and compared this with studies from other gorilla populations, as well as other great apes. Their summary demonstrates the potential for variation in context with regard to gorilla chest beating ranging from play, displays, to sexual initiation (Salmi & Muñoz, 2020). Similarly, hand clapping has been documented in both play and alert contexts, although the former primarily in captive gorilla populations (Salmi & Muñoz, 2020). Nonetheless, such a detailed comparison demonstrates the potential to unveil further cultural communication by considering variation in the behavioral context or function of gorilla signals across groups.

### 2.4 Bonobos (*Pan paniscus*)

Although bonobos are perhaps the great ape with the least amount of observational hours collectively, variation among groups within the Democratic Republic of Congo has been documented for decades, primarily involving nonforaging tool use behaviors (Hohmann & Fruth, 2003). Many of the bonobo behaviors that demonstrate potential for cultural variation are behaviors that are also observed in chimpanzees (Hohmann & Fruth, 2003). Hohmann and Fruth (2003) first documented and compared bonobo behaviors at the study site of Lomako, with behaviors known to be cultural in chimpanzees, and compared descriptions and contexts with those reported for Wamba and other bonobo populations. Their approach highlighted potential cultural variation in some communicative gestures such as leaf clipping and hand‐clasping. More recently, Samuni et al. (2022) provided a detailed repertoire of multiple bonobo communities of Kokolopori, adding another bonobo population that could be compared for within‐species cultural variation. Samuni and colleagues reported that whilst all three populations, Wamba, Lomako, and Kokolopori drag branches during displays and take an object in the mouth or hand to initiate play, other object gestures, such as leaf clip and drop twig, occur in some communities and not others, clearly documenting cultural variation in the communicative domain. Similar findings were reported by Badihi et al.'s (2023) survey of leaf‐modifying gestures. What remains to be examined is potential variation in meaning or context for the same signal across these different bonobo communities, and the exact action sequences or gestural forms used to produce these signals across groups. Additionally, bonobo vocalizations remain an understudied area of research in the wild although initial findings report flexibility in regard to call combinations (Schamberg et al., 2016). Cross‐population comparisons of vocal repertoires and variability may reveal new insights into bonobo cultural communication.

## 3 WHAT WE DON'T KNOW: OUTLOOK AND PROSPECTS FOR FUTURE STUDIES

The majority of cultural communicative behaviors described thus far are unique to each of the four major great ape taxa except for a handful of behaviors that are found in at least two great ape species, namely the raspberry, handclasp, leaf clip and leaf drop (Table 1). Furthermore, the vast majority of gestural signals reported as group‐specific, cultural traits across all four great ape taxa are auditory gestures (except handclasp and leaf drop), meaning they are gestures that produce sound via interaction with the body, an object, or a tool. This is notable given that the interaction with another object or tool could permit greater flexibility and innovation with respect to signal form and may therefore enable group‐specific variants to emerge. Lameira et al. (2012) refer to these types of gestures as “acoustic gesture‐calls” and describe the orangutan kiss squeak as the sole example of an “instrumental gesture‐call” whereby an object or tool is used to modify sound produced from the oro‐laryngeal cavity (Lameira et al., 2012). Irrespective of what they are called, these auditory gestures appear to dominate reports of culturally varying gestural signals and therefore should be given special attention in future studies investigating cultural forms of great ape communication.

**Table 1:** Great ape communicative signals describing cultural variation in the wild and those that show great potential but require further research.

	Cultural variation described		Potential for cultural variation	
	Vocal	Gestural	Vocal	Gestural
**Chimpanzee**	*Pant hoots* (Arcadi [1996]; Crockford et al. [2004]; Desai et al. [2022]; Girard‐Buttoz et al. [2022]; Mitani et al. [1999]; Mitani et al. [1992])	*Leaf clip* (Badihi et al. [2023]; Bessa et al. [2022]; Hobaiter and Byrne [2014]; Kalan and Boesch [2018]; Luncz and Boesch [2015]; Nishida [1980]; Sugiyama [1981]; Watts [2007]), *hand clasp* (McGrew et al. [2001]; Nakamura and Uehara [2004]; Piel et al. [2017]; Pika and Deschner [2019]; Whiten et al. [1999]) *rain dance* (Hashimoto [1998]; Whiten et al. [1999]; Whiten et al. [2001]), *knuckle knocking* (Cissewski and Luncz [2021]), *leaf drop, leaf tear* and *leaf pull* (Nishida [1980])	*Food calls* (Bouchard and Zuberbühler [2022]; Fedurek and Slocombe [2013]; Kalan and Boesch [2015]; Slocombe et al. [2010])	*Buttress drumming* (Arcadi et al. [1998]; Boesch [1991])
**Orangutan**	*Long calls* (Delgado et al. [2008]; Krützen et al. [2011]; van Schaik et al. [2013]), *raspberries* (van Schaik et al. [2003]; Wich et al. [2012]), *nest smacks* (Wich et al. [2012])	*Kiss squeak* (Hardus et al. [2009]; Lameira et al. [2013])		
**Gorilla**		*Tree slap* (Robbins et al. [2016])	*Raspberry* (Robbins et al. [2016])	*Tapping head, hand clapping* and *chest beating* (Robbins et al. [2016]; Salmi and Muñoz [2020])
**Bonobo**		*Leaf clip* (Hohmann and Fruth [2003]; Samuni et al. [2022]), *twig/leaf drop* (Samuni et al. [2022])		*Hand clasp, leaf strip* and *buttress drumming* (Hohmann and Fruth [2003])

This review of within and across species variation in great ape vocal and gestural signals clearly demonstrates that there is substantial variation in the presence or absence of signals from a particular group's repertoire, whilst not always sufficient information on variation in context and meaning, or vice versa. Crucially, both aspects can be shaped by social learning: the production and *know‐how* to create and use a signal as well as the understanding, or *know‐what* a signal means within a social group (i.e., its function or meaning; or perhaps *know‐why*). Many of the behaviors documented in this article require further research on one or both of these aspects, which will contribute to a more holistic perspective into cultural forms of communication in great apes and, importantly, will contribute substantially to our understanding of the evolutionary origins of cumulative culture and symbolism.

**I**. **The Emergence of Cumulative Culture**
  Cultural communication in great apes has been documented in both vocal and gestural domains. While this notion remains a relatively underrepresented form of culture in current debates surrounding the evolution of cumulative culture (Whiten, 2022), it has at the same time garnered considerable support from primatologists researching great apes in the wild (Gruber & Biro, 2023; Koops et al., 2023). Thus far, the strongest evidence for cumulative culture in nonhuman primates arguably comes from decades of observations collected on the food washing behaviors of Japanese macaques (Schofield et al., 2018). Here, historical, longitudinal data clearly document the emergence of more complex, varied food washing behaviors over time that spread within Japanese macaque troops. Other compelling examples of potential cumulative culture have been proposed for the variable termite fishing techniques employed by different chimpanzee communities to extract termites from both underground and above ground termite nests using stick tools (Boesch et al., 2020). The nuanced variation in body positions, arm and wrist support for fishing sticks, and the different techniques for ingesting the termites (e.g., directly from the stick, wiping with the hand first, etc.) demonstrate a combination of techniques that are more likely to be shared within a chimpanzee community than across communities, suggesting some degree of social convergence or conformity in termite fishing techniques (Boesch et al., 2020). This detailed ethnographic approach of deconstructing a behavior is notable as this is exactly how we are able to isolate the *know‐how* (action forms and techniques) from the *know‐what* (end‐goal) or *know‐where* (ecological conditions) of socially learned behaviors. As skeptics of animal cultures have argued, *know‐how* is the type of knowledge that we expect to be most amenable to high‐fidelity social learning, where we might predict the emergence of group conformity and cumulative culture (Tennie et al., 2020).
  Hence, the study of cultural forms of communication which inherently investigates variation in signal forms, that is, the ways in which the body, face, mouth, tongue, or vocal cords might interact to create countless ways of information transfer, has much to contribute to the study of *know‐how* social transmission. As described above, detailed analyses of vocal signatures have revealed some support for vocal dialects (Crockford et al., 2004; Desai et al., 2022), while detailed analyses of leaf‐modifying behaviors are further revealing the potential for dialects in the gestural domain (Badihi et al., 2023). Considering that great apes often employ other objects into their communicative displays, such as sticks and stones (Goodall, 1971), we may further expect to find evidence of group‐specific dialects in regard to these behaviors.
  The use of stones in communicative contexts may offer particularly novel insight with regard to the emergence of cumulative culture. In chimpanzees, the AST behavior is accompanied by the pant hoot vocalization and is sometimes observed in conjunction with buttress drumming. Both pant hoots and buttress drumming are universal behaviors while AST is not. Detailed analyses have shown group‐specific variation in both pant hoots and buttress drumming that has been described as socially learned (Arcadi et al., 1998; Crockford et al., 2004; Mitani et al., 1992; see above for discussion of these studies). If we consider then that male chimpanzees have been reported to incorporate objects into their displays, such as throwing sticks or stones, but that AST is something unique given that the rock is thrown in an aimed fashion at a tree (Kalan et al., 2019), we may see AST as representing some increasingly complex, emergent form of a male display that has become a habitual behavior in a select number of chimpanzee communities. Moreover, AST can be reduced into its component behavioral units, arguably representing an increase in complexity given the increased motor‐coordination necessary for the aimed throw and tool use (Kalan et al., 2019). For some, this would satisfy definitions of cumulative culture, “a modification (change in the sequence or form of behavioral elements) of a cultural trait (i.e., acquired via social learning) that enhances its complexity, efficiency, security or convenience” (Schofield et al., 2018), or even the more stringent human‐specific definitions of cumulative culture which require a progressive increase in complexity (Dean et al., 2014). Still, AST may be within the possibilities for a single individual to invent and therefore may not be a culturally dependent trait (Tennie et al., 2020). However, it may be argued that the persistence of AST sites in the landscape indicates that they are culturally dependent in that a single individual is unlikely to be able to sustain this communicative behavior without it having meaning and utility for others in the group with whom it is communicating. Given that we have not been able to observe the innovation and emergence of AST, but can only rely on deconstructions of the behavior in hindsight, the potential cumulative cultural aspects of AST remain to be tested. Nevertheless, the idea that complex, communicative signals may be composed of discrete behavioral units where social and cultural behaviors can be easily integrated or added on, is not new when we consider the cultural evolution of bird and whale songs (Aplin, 2019; Whitehead & Rendell, 2015 but see also Mercado, 2022). This is yet another reason why the communication realm may provide us with much further insight into the extent of cultural complexity in great apes.

**II**. **The Emergence of Symbolism**
  Humans are a “symbolic species” (Deacon, 1997) and symbolism influences every aspect of human life, including how we think, perceive the world, and most importantly how we communicate: in other words, language (Deacon, 1997; Noble & Davidson, 1996). Symbolic communication is defined by an arbitrary signal form and socially transmitted meanings or cultural conventions (Boesch, 1991; Deacon, 1997). In captivity, nonhuman great apes have demonstrated significant abilities to learn and use symbolic systems to communicate with humans and even other trained, enculturated apes (Gardner et al., 1989; Savage‐Rumbaugh et al., 1993). However, we know little about their natural capacity for symbolism despite some wild populations exhibiting potentially cultural forms of communication as described in this paper. We therefore argue that an in‐depth investigation into cultural communication is the key to understanding the symbolic capacity of wild great apes since only these behaviors have shown any degree of arbitrariness and group‐specificity, rather than species universals. Recently, Watson et al. (2022) proposed an optionality framework where the variation in mapping between signal and function across social groups of animals could be interpreted as emerging evidence of arbitrariness in nonhumans. Such a comparative framework demonstrates the potential for these abstract, human‐specific terms to be examined from a nonhuman, nonlanguage lens. It is within the interest of comparative research that we may also consider that cultural forms of communication, if deemed arbitrary, would satisfy definitions of symbolic communication. Indeed, longitudinal observations of group‐specific gestural variants, such as leaf‐clipping (Kalan & Boesch, 2018), have recently been described as “symbolic signal use” (Cissewski & Luncz, 2021), as well as a notable case study of buttress drumming by a single male chimpanzee in the wild (described above, Boesch, 1991). Alongside arbitrariness, displaced reference, or the ability to transmit information about “things that are remote in space or time (or both)” (Hockett, 1960, p. 90), is also taken to be a hallmark of symbolic communication and thus language. While rare in nonhuman primates, displaced reference has been observed in wild orangutan mothers' reactions to predator models (Lameira & Call, 2018). Recently, AST sites in wild chimpanzees have been suggested to be potentially symbolic, not from their use per se but rather the potential meaning or significance they may represent on the landscape (Kalan et al., 2019; Kühl et al., 2016) where these sites may be interpreted as referring to time‐displaced communicative events. Displaced reference is therefore an area of research that should be explored further for its potential relevance for the evolutionary origins of symbolism.

## 4 CONCLUSION

We are at an exciting new point for field researchers studying wild great apes. Leveraging longitudinal datasets from long‐term field sites, and new methods for studying unhabituated wild apes (Hansen et al., 2022) can provide new data to tackle questions about the evolution and complexity of cultural forms of communication. As summarized in this paper, the last decades of research have primarily shown group‐specific variation in communicative signals by comparing a handful of populations at most but there is great potential for expanding these comparisons more broadly and systematically. In particular, data sharing across sites and across species would enable a greater breadth of comparative research into great ape cultural communication. Robust inferences will depend upon comparable research protocols across populations which will require some degree of cross‐site, cross‐species collaboration. Hence, at the moment data on great ape cultural communication in the wild appears to be growing but will be vastly enriched with systematic comparisons of the same behavior within and across species. In addition, variability in both culture and communication are integral to the social lives of great apes and can help to predict behavioral flexibility in the face of changing environments due to anthropogenic pressures (Kalan et al., 2020; Kühl et al., 2019). Such research efforts would also align well with recent calls to include animal cultures into conservation efforts (Brakes et al., 2019; Carvalho et al., 2022; Whiten, 2021) expanding the relevance of this work beyond academia.

## AUTHOR CONTRIBUTIONS

**Ammie K. Kalan**: Conceptualization (lead); data curation (lead); Investigation (lead); project administration (lead); supervision (lead); Writing—original draft (lead); writing—review and editing (lead). **Robyn Nakano**: Data curation (supporting); investigation (supporting); writing—original draft (supporting); writing—review and editing (supporting). **Lindsey Warshawski**: Data curation (supporting); investigation (supporting); writing—original draft (supporting); writing—review and editing (supporting).

## CONFLICT OF INTEREST STATEMENT

The authors declare no conflict of interest.

## Data Availability

Data sharing is not applicable to this article as no new data were created or analyzed in this study.
